# Cardiogenic Shock and Guillain–Barré Syndrome as the First Manifestations of Pheochromocytoma

**DOI:** 10.1155/2021/6691095

**Published:** 2021-06-03

**Authors:** Maryam Heidarpour, Nafiseh Sereshti, Davood Shafie, Bijan Iraj, Hassan Rezvanian

**Affiliations:** ^1^Isfahan Endocrine and Metabolism Research Center, Isfahan University of Medical Sciences, Isfahan, Iran; ^2^Heart Failure Research Center, Isfahan Cardiovascular Research Institute, Isfahan University of Medical Sciences, Isfahan, Iran

## Abstract

Febrile congestive heart failure is a rare first manifestation of pheochromocytoma. Herein, the case of a 31-year-old female with febrile congestive heart failure and subsequent cardiogenic shock is presented. After intensive care unit (ICU) admission and further evaluating the right adrenal mass observed in abdominal ultrasonography, the diagnosis of pheochromocytoma was confirmed. Then, she was scheduled for the right adrenalectomy. Before surgery, she complained of acute-onset progressive muscle weakness in the lower limbs, followed by the upper limbs. After further investigation, she was diagnosed with Guillain–Barré syndrome and treated with intravenous immunoglobulin (IVIG). She recovered well after the right adrenalectomy, and during the subsequent 18 months, the follow-up did not reveal any complications, and left ventricular function recovered to normal.

## 1. Introduction

Pheochromocytoma is a rare neuroendocrine tumor with the ability to synthesize and overproduce catecholamines, developed in the adrenal medulla [1, 2]. Its clinical presentation is highly variable, but the classic triad is headache, sweating, and palpitation [3]. Less often, the tumor will cause severe cardiovascular complications such as myocardial infarction, arrhythmias, pericardial effusion, and congestive heart failure. Pheochromocytoma-induced cardiomyopathy is similar to Takotsubo cardiomyopathy and myocarditis [4, 5]. Diagnosis of pheochromocytoma-related cardiomyopathies is often delayed because of the atypical presentation. Given the potential reversibility of the cardiomyopathy, early diagnosis and resection of the pheochromocytoma are critical, while delayed diagnosis may lead to irreversible cardiac remodeling and death [6].

On the other hand, Guillain–Barré syndrome (GBS) is an acute immune-mediated polyradiculoneuropathy. Symmetrical limb weakness and hyporeflexia are typical manifestations of the GBS. However, half of these patients present with cranial nerve involvement. There have been few case reports of cardiovascular involvement in patients with GBS [7–10]. Here, we describe a case of pheochromocytoma presented as febrile congestive heart failure with subsequent cardiogenic shock and GBS. Finally, we discuss our diagnostic and management insights and report the patient's condition over 18-month follow-up period.

## 2. Case Presentation

**Figure 1:**
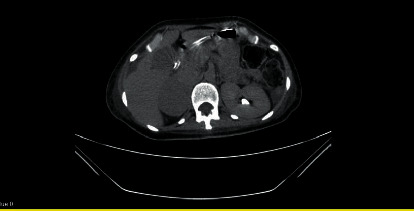
Abdominal CT scan of the patient shows heterogeneous right adrenal mass.

A 31-year-old female presented to the emergency room with severe dyspnea, palpitation, acute generalized abdominal pain, and vomiting without blood or bile in February 2019. Upon reviewing systems and past medical and surgical history, she admitted to a 5 kg weight loss over the past month. She had a cesarean section 18 months ago without any complications. She had no history of hypertension (HTN), diabetes mellitus (DM), and cigarette smoking. Her drug history was negative, especially for illegal drugs, such as methamphetamines and cocaine. Her symptoms started 5-6 hours before admission. Initial vital signs included a blood pressure of 80/50 mmHg, heart rate of 130 beats per minute (bpm), respiratory rate of 30 per minute, O_2_ saturation of 81% on room air, and temperature of 38.1°C. Physical examination revealed diffuse bilateral crackles but no decreased breath sounds at the base. Generally, the patient appeared confused and unstable. During the initial physical examination, she had a cardiac arrest, and cardiopulmonary resuscitation was done for 10 minutes, and it was successful. After that, she was sent to the intensive care unit (ICU). At this time, ECG showed sinus tachycardia, and transthoracic echocardiogram (TTE) demonstrated severe left ventricular (LV) systolic dysfunction with ejection fraction (EF) of 10%, normal LV size, mild to moderate mitral regurgitation, mild tricuspid regurgitation, and moderate pericardial effusion. Laboratory data are shown in Table 1. She received norepinephrine (starting infusion rates of 0.025 *μ*g/kg/min), meropenem (500 mg IV every 8 hours), and ciprofloxacin (500 mg IV every 12 hours). On the fourth day, norepinephrine was discontinued, and hemodynamic stability was maintained even after discontinuation of vasopressor support. However, her fever continued to peak at 39°C. Tracheal aspirate, blood, and urine cultures were taken, and abdominal ultrasonography was performed due to non-specific abdominal discomfort. Ultrasonography showed a well-defined hypoechoic mass (44 × 57 mm) with internal vascularity in the right adrenal gland. In the next step, on the seventh day, abdominal computed tomography (CT) and catecholamine urine tests were requested for suspicion of an adrenal tumor. Abdominal CT showed a heterogeneous right adrenal mass measuring 47 × 54 mm (Figure 1). The adrenal mass on the unenhanced images had a density of 36 Hounsfield units. Laboratory values were notable for a 24-hour urine metanephrine of 2510 *µ*g/day (normal  <315 *µ*g/day), normetanephrine of 8657 *µ*g/day (normal  <670 *µ*g/day), adrenaline of 386 *µ*g/day (normal  < 20 *µ*g/day), noradrenaline of 1044 *µ*g/day (normal  < 90 *µ*g/day), and vanillylmandelic acid of 70 mg/day (normal  < 13.6 mg/day). Indeed, all cultures were negative for infectious organisms. These findings were consistent with the diagnosis of pheochromocytoma. She was started on phenoxybenzamine 10 mg twice a day, and the dosage was increased by 10 mg every three days to a maximum dosage of 20 mg twice a day. On the tenth day, she complained of acute-onset progressive muscle weakness in the lower limbs, followed by the upper limbs. The muscle strength examination showed severe weakness in four limbs with a Medical Research Council (MRC) scale of 1/5 in proximal and 2/5 in distal of the upper extremities and 2/5 in proximal and 2/5 in distal of the lower limbs. She had no spine sensory level. Deep tendon reflexes were absent. Upper motor neuron disorders and meningeal irritation signs were absent. Lumbar puncture for cerebrospinal fluid (CSF) analysis was performed, and study results are shown in Table 2. Brain magnetic resonance imaging (MRI) was done and showed a normal finding except for a few foci of cortical and subcortical infarction. Electromyography (EMG) and nerve conduction velocity (NCV) showed acute predominantly motor axonal polyneuropathy. Therefore, the diagnosis of GBS was made, and the patient was treated with intravenous immunoglobulin (IVIG) 2 g/kg divided over five days. After that, muscle weakness gradually improved. On the fourteenth day, propranolol was prescribed with a dose of 10 mg every 8 hours. Then, the right adrenalectomy was performed without any surgical complications, and the postoperative histopathologic report was in agreement with a diagnosis of pheochromocytoma. No complications were reported in the postoperative period. Predischarge TTE showed LVEF of 40%, normal LV size, mild mitral regurgitation, mild tricuspid regurgitation, and no pericardial effusion. She was followed in an outpatient setting regularly. Two weeks after adrenalectomy, the patient had no evidence of the disease. Laboratory values were notable for a 24-hour urine metanephrine of 107 *µ*g/day (normal  <315 *µ*g/day), normetanephrine of 210 *µ*g/day (normal  <670 *µ*g/day), adrenaline of 15 *µ*g/day (normal  < 20 *µ*g/day), noradrenaline of 34 *µ*g/day (normal  < 90 *µ*g/day), and vanillylmandelic acid of 7.1 mg/day (normal < 13.6 mg/day). Today, 18 months after hospital discharge, her general condition is good, and the catecholamine urine test results are negative for pheochromocytoma. The latest echocardiography demonstrates the LVEF to be 50%, and other echocardiographic measurements are within the normal values.

## 3. Discussion

This case report presented a young female with complicated pheochromocytoma having unusual presentations of hypotension, cardiac arrest, cardiomyopathy, and fever. Also, she was complicated with GBS in the course of hospitalization.

Pheochromocytoma exhibits variable characteristics with diverse presentations. Although it is rare, it is a fatal emergency, and diagnosis can be extremely challenging. Since our case presented predominantly with cardiac problems, it is mandatory to discuss cardiovascular complications of pheochromocytoma in detail. Pheochromocytoma usually manifests as severe hypertension, circulatory shock, arrhythmias, myocardial injury, and cardiomyopathy—the excessive catecholamine exposure of myocardium results in toxic effects like myocardial necrosis and cardiomyopathy [11]. The prevalence of cardiomyopathy in pheochromocytoma has been reported to be about 11% [12]. Of course, we must also consider cases of pheochromocytoma that have not been diagnosed and reported. In these patients, cardiomyopathy may be presented as a global or focal abnormality. Interestingly, a complete reversal of pheochromocytoma cardiomyopathy has been reported in a short period of as much as eight days, following the tumor's removal [13].

Approximately 50% of patients with pheochromocytoma have a hypertensive crisis; however, 5–15% are normotensive, and in rare instances, these patients have hypotension. Bergland et al. reported in a review of 539 cases of pheochromocytoma that 2% of the cases presented with hypotension and shock [14]. Possible hypotension mechanisms in pheochromocytoma are hypovolemia, desensitization of blood vessels to catecholamine, reduced vascular resistance due to high epinephrine release from the tumor, myocardial contractile dysfunction, and hemorrhagic necrosis of the tumor [14].

Fever is also an uncommon presentation in these patients. In a case series of 25 patients, 12% presented with fever [15]. The excessive release of epinephrine has been postulated as a cause of fever by combining hypermetabolism and cutaneous vasoconstriction [16]. Of course, infectious causes and inflammatory disease should always be considered in all patients with febrile pericarditis and it should be evaluated first.

The further exciting twist in our case is the co-occurrence of GBS and pheochromocytoma. Although this is not impossible, it rarely happens. The average annual incidence of GBS was 1 per 100 000 patient-years, while the incidence of pheochromocytoma are approximately 0.8 per 100 000 patient‐years [5, 17]. Abdel-Salam et al. reported the case of GBS with subsequent pheochromocytoma [17].

Indeed, Ahmad et al. reported that urine and plasma catecholamine levels were elevated in patients with GBS who had autonomic instability [18]. However, in our case, the clinical manifestations of GBS occurred after the diagnosis of pheochromocytoma, and cardiac complications began to improve after adrenalectomy. Although the exact cause of GBS remains to be clearly understood, previous studies indicated an association between immunological stressors (such as viral infection and vaccination) and GBS. Our case denied any flu-like symptoms or vaccination in the preceding two weeks. Therefore, the question arises that can hypersecretion of catecholamine and pro-inflammatory states in pheochromocytoma provoke GBS?

In our case, considering the young age, female sex, fever and dyspnea as first manifestations, normal ECG except for sinus tachycardia, lack of traditional cardiovascular risk factors, and negative drug history for cocaine, coronary heart disease was less suspected.

Fortunately, the patient was diagnosed and treated at a referral center despite having a large tumor and catastrophic symptoms and signs. Finally, we emphasize that the clinical manifestations of pheochromocytoma depend on the various hormone secretion, and the diversity of this matter should be considered in any clinical scenario. However, whether there is a direct link between GBS and pheochromocytoma is a mystery that needs to be answered with more detailed studies.

## Figures and Tables

**Table 1:** Laboratory test results.

Test	Day 1	Day 10	Normal values
FBS	104	109	Up to 100 mg/dl
BUN	26	88	7–20 mg/dl
Cr	1.3	1.6	0.8–1.3 mg/dl
Na	138	152	135–145 mEq/L
K	3.7	3.3	3.5–5 mEq/L
Ca	8.4	7.8	8.5–10.5 mg/dl
Ph	4.6	3.9	3.4–4.5 mg/dl
Mg	2.2	1.7	1.7–2.2 mg/dl
Troponin	17.9	54	Up to 0.4 ng/ml
D-Dimer	5794	—	Up to 500 ng/ml
TSH	1.4	—	0.35–0.5 mIU/L
WBC	30.9	29.0	4.5–11 × 10^9^/L
Neut	82.4%	91%	55–70%
Lymph	7.6%	4%	20–40%
HB	10.1	9.8	12–15 g/dl
aPTT	43	35	30–40 msec
INR	1.2	1.5	Up to 1.1
AST	401	390	5–40 IU/L
ALT	535	912	7–56 IU/L
ALP	306	342	20–140 IU/L
ESR	1	2	Up to 29 mm/hr
CRP	13	47	Up to 10 mg/L
Alb	3.3	3.2	3.3–5.4 g/dl

BUN: blood urea nitrogen; Cr: creatinine; Na: sodium; K: potassium; Ca: calcium; Ph: phosphorus; Mg: magnesium; TSH: thyroid-stimulating hormone; WBC: white blood cell; Neut: neutrophil; Lymph; lymphocyte; HB: hemoglobin; aPTT: activated partial thromboplastin time; INR: international normalized ratio; AST: aspartate aminotransferase; ALT: alanine aminotransferase; ALP: alkaline phosphatase; ESR: erythrocyte sedimentation rate; CRP: C-reactive protein; Alb: albumin.

**Table 2:** CSF analysis results.

Test	Patient's values	Normal values
CSF glucose	68	>50% serum
Serum glucose	118	Up to 200 mg/dl(random sampling)
Protein	0.48	0.15–0.45 g/L
WBC	0	<5 (mm^3^)
RBC	3	0 (mm^3^)
Viral PCR (HSV 1 and 2, VZV, and Enterovirus)	Negative	Negative

CSF: cerebrospinal fluid; WBC: white blood cell; RBC: red blood cell; HSV: herpes simplex virus; VZV: varicella-zoster virus.

## Data Availability

No data were used to support this study.
